# Volcanic Mud Incorporating Paper Sludge as Raw Materials for Lightweight Aggregates

**DOI:** 10.3390/ma12091511

**Published:** 2019-05-09

**Authors:** Chung-Ho Huang, Hao-Yu Fang, Han Chen

**Affiliations:** Department of Civil Engineering, National Taipei University of Technology, No.1, Sec. 3, Zhongxiao E. Road, Da’an District, Taipei City 106, Taiwan; chiwei0720@gmail.com (H.-Y.F.); cbpl2001@yahoo.com.tw (H.C.)

**Keywords:** lightweight aggregate, waste reuse, sintering, crushing strength

## Abstract

This study assessed the use of volcanic mud collected from southern Taiwan and the incorporation of paper sludge for manufacturing lightweight aggregate (LWA). The firing process of the raw materials and related sintering mechanisms, including sintering temperature and time, were investigated. LWA was manufactured at sintering temperatures ranging from 950 to 1275 °C with soaking times from 2 to 15 min, and preheating temperatures ranged between 500 and 700 °C with soaking times from 5 to 15 min. Using volcanic mud and mixed sludge (volcanic mud with added paper sludge) resulted in the successful manufacture of various qualified LWAs with particle density ranging from 973 to 1950 g/cm^3^, water absorption from 6.2 to 20.0%, and crushing strength from 2.2 to 15.8 MPa.

## 1. Introduction

Improving resource efficiency is a considerable challenge in modern society. One aspect of the solution to this problem is the reuse of waste materials as commercially valuable materials. The synthetic lightweight aggregate (LWA) industry has a long record of reusing natural materials and waste from other industries, such as sedimentary and low-grade metamorphic rocks (e.g., clays, shale, and tuffs) [1,2,3,4] and sludge (e.g., sewage sludge, water treatment sludge, and municipal sludge) [5,6,7,8]. These materials have been applied in LWA manufacturing as either raw materials in the kiln feed or as a partial replacement for raw materials.

Mud volcanoes are landforms created by the eruption of mud or slurries, water, and gases. The mud produced by mud volcanoes is mostly formed as hot water deep below the Earth’s surface, which mixes and blends with subterranean mineral deposits to create the mud slurry exudate and is then forced upward to the surface through a geological fault or fissure. Because the solid substance of the ejected mud mostly consists of shale, clay, sand, and silt, it possesses potential for manufacturing LWA. 

Numerous mud volcanoes are located in southern Taiwan, such as in Pingtung County and Kaohsiung County. The disposal of the dried mud requires careful consideration for it to be managed in an environmentally acceptable and sustainable manner. To date, volcanic mud has primarily been disposed of using landfilling. However, landfill disposal is not ideal and involves increasingly strict legislation and increasing costs [9]. This situation provides increasing incentives to develop alternative and economically viable reuse and recycling options. These alternatives include the use of volcanic mud in building material and construction, such as the use of the mud in mortar and concrete, including controlled low-strength material and ready-mixed soil material [10,11,12,13,14,15]. Other studies have proposed manufacturing LWA from reservoir sediment, expanded clay, and zeolitic tuffs [16,17,18,19]. Because the main constituents of volcanic mud are inherently similar to those of shale, clay, and reservoir sediment, it is worthwhile to investigate the technology for manufacturing LWA by using volcanic mud as raw material. Because of the large amount of volcanic mud in Taiwan and lack of an effective reuse method, this study investigated the feasibility of using the mud as a raw material for LWA.

Paper sludge is another type of waste. Currently, sludge in Taiwan is generally either deposited or burned. Disposal of this sludge is a critical environmental and economic problem for the paper industry. Paper sludge mainly consists of high organic matter and inorganic compounds, such as clays and calcium carbonate. Converting the paper sludge into an environmentally benign material has been proposed by incorporating it into a ceramic body [20]. Use of this sludge for purposes such as glass ceramics [21], lightweight porous and high-strength composite materials [22], and an organic pore-forming agent in bricks [23] has also been reported. Because some of the components of the paper sludge (e.g., calcium compounds and iron) contain the chemical elements considered in the so-called fluxing parameter (fluxing = CaO + MgO + NaO + K_2_O + MgO; Riley [24]), their presence may influence the softening and melting temperatures as well as the bloating of LWAs.

Taiwan produces more than 500,000 t of paper sludge every year, and the accumulated stockpiled amount of the waste is estimated to be over 2,000,000 t. Most of this paper sludge was disposed of in landfills during the 2010s. However, locating suitable sites for sludge disposal has become increasingly difficult. Therefore, researching and developing appropriate means for reusing this paper sludge beneficially is urgent. The aforementioned manufacturing techniques motivated this investigation to appraise the feasibility of paper sludge as a mineral addition or an organic pore-forming agent for LWA production. 

## 2. Experimental Procedure

### 2.1. Materials

**Figure 1:**
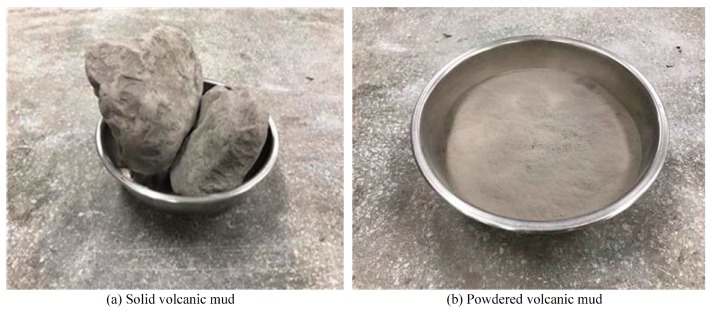
Volcanic mud.

**Figure 2:**
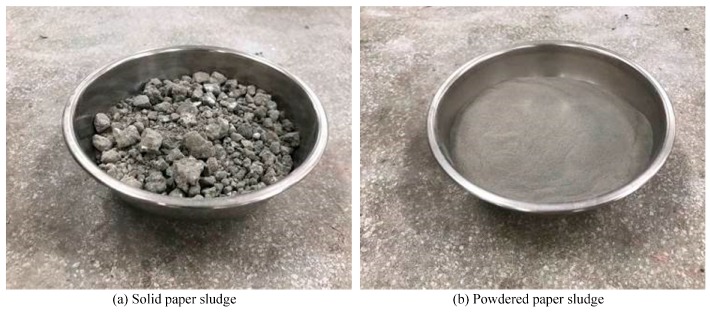
Paper sludge.

In this study, volcanic mud and paper sludge were used. Volcanic mud was selected as the sole raw material for manufacturing LWA, whereas paper sludge was used as a partial replacement of the volcanic mud for LWA production. The dried solidified volcanic mud (Figure 1a) was collected from farmland in Pingtung County, Taiwan. This solid mud was jaw crushed, milled, and screened into fractions with sizes of under 75 μm (Figure 1b). The paper sludge was obtained from a paper factory (Chung Hwa Pulp Corporation) in Hualien County, Taiwan. The received solid sludge (Figure 2a) was initially subjected to pretreatment, such as drying (105 °C for 24 h) and grinding (Figure 2b). The obtained products were ground and sieved to a particle size of under 75 μm.

### 2.2. Characteristic Analysis of Volcanic Mud and Paper Sludge

The raw materials after grinding and screening pretreatment were physically and chemically characterized to evaluate their feasibility for LWA manufacturing.

#### 2.2.1. Physical Characteristics

**Figure 3:**
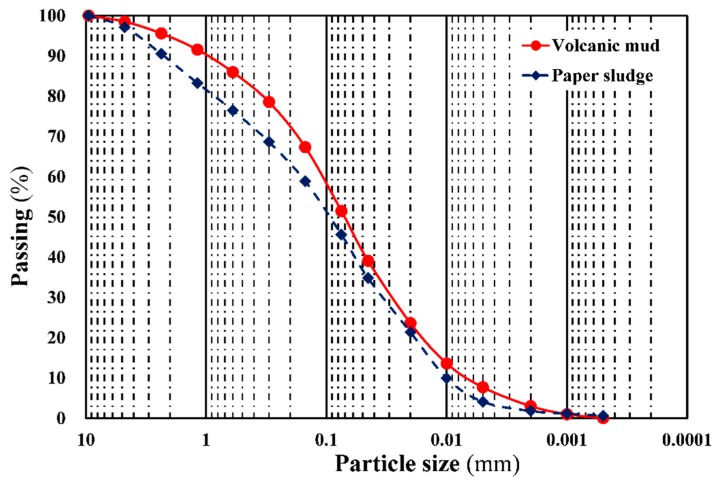
Particle size distribution of raw material.

The physical tests of the volcanic mud and paper sludge samples included particle size analysis, specific gravity, and soil classification. The test methods for sieve analysis, specific gravity, and soil classification were carried out in accordance with ASTM D421, ASTM D854, and ASTM D2487, respectively. Table 1 presents the test results for the samples. The volcanic mud samples had an average specific gravity of 2.73, which was similar to that of the general soil, whereas the paper sludge samples had a smaller average specific gravity of 1.03 because of their organic matter content. The Atterberg limit test is a basic measure of the critical water content of fine-grained soils. The tests include shrinkage limit (SL), plastic limit (PL), and liquid limit (LL), which are outlined in ASTM D4943. The plasticity index (PI) is a measure of the plasticity of a soil. The PI is the size of the range of water contents where the soil exhibits plastic properties, that is, the difference between the liquid limit and the plastic limit (PI = LL-PL). Soils with a high PI tend to be clay, those with a lower PI tend to be silt, and those with a PI of 0 (non-plastic) tend to have little or no silt or clay. From the test results, the plasticity index of the volcanic mud and paper sludge samples ranged from 8–10% and 10–15%, respectively. According to the Unified Soil Classification System (USCS), both materials were classified as clay of low to medium plasticity. In addition, Figure 3 shows the particle size distribution of the samples. The particle size of both material samples ranged mainly from 1.5 to 75 μm, and the proportion passing the 75 μm (no. 200) sieve was more than 50%. This indicated that both materials can be classified as silt or clay.

#### 2.2.2. Chemical Characteristics

**Figure 4:**
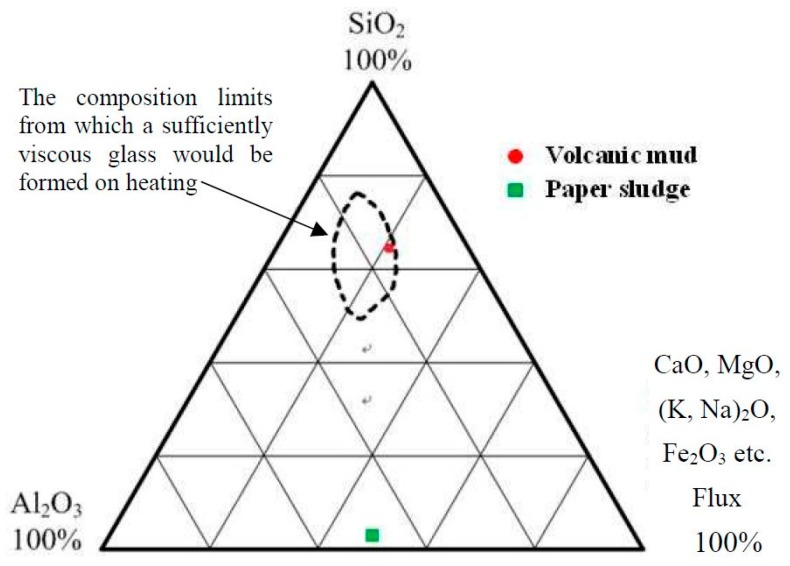
Ternary diagram of bloating materials after Riley.

Volcanic mud and paper sludge were analyzed chemically using an energy-dispersive X-ray fluorescence spectrometer (Thermo Fisher Scientific Taiwan Co. Ltd., Taipei, Taiwan). Table 2 shows the analysis results. The analysis revealed that the volcanic mud comprised mainly SiO_2_, Al_2_O_3_, and Fe_2_O_3_, in which the values of fluxing (Fe_2_O_3_, CaO, K_2_O, MgO, and Na_2_O) reached approximately 14%, ensuring the development of a high-temperature glassy phase with sufficient viscosity. By plotting the analyzed results in Riley’s ternary diagram, the composition of volcanic mud was determined to be a fit bloating material for manufacturing LWA (Figure 4). However, the analyzed paper sludge indicated CaO as a main component (41.27%), and was associated with minor silica (3.55%), magnesia (5.57%), and ferric oxide (2.20%) (Table 2). A relatively high content of organic matter in the paper sludge yielded a considerable loss of ignition at 38.54%. Plotting the chemical compositions of the paper sludge in Riley’s ternary diagram (Figure 4) indicated that the composition was not bloating material because of the lower concentration of SiO_2_ and Al_2_O_3_. These results indicated that only the volcanic mud was feasible for manufacturing LWA.

Fluxing may help lower a material’s melting point [25,26]. The greater the content of fluxing, the lower the temperature is when the material starts to soften. This is due to the fact that the chemical composition of paper sludge contains relatively greater amounts of CaO, MgO, and Na_2_O. Therefore, incorporating the paper sludge into volcanic mud may be an effective method to lower the softening temperature of the pellets when manufacturing LWA.

#### 2.2.3. Mineralogical Characteristics

**Figure 5:**
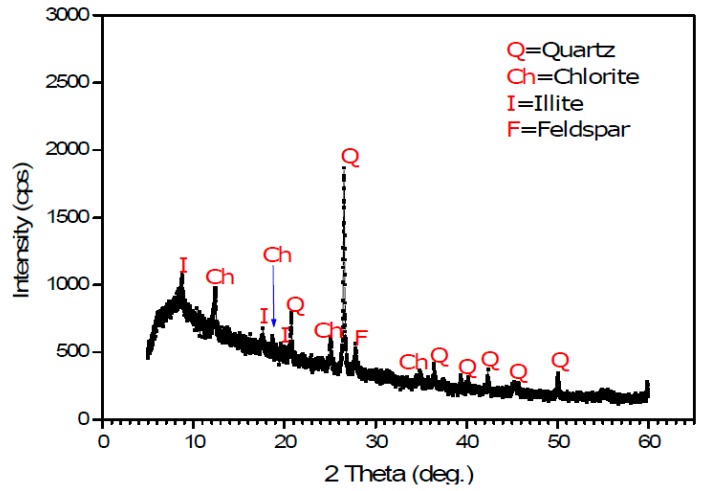
X-ray diffraction pattern of the volcanic mud.

Raw volcanic mud material was also characterized mineralogically through X-ray diffraction. As shown in the typical diffraction diagram (Figure 5) of the volcanic muds, quartz (Q) peaks were dominant and notable, followed by chlorite (Ch), illite (I), and feldspar (F) peaks. These results were consistent with the mineralogy of expanded clay and expanded shale [17,18,19]. Consequently, the volcanic mud appeared to have potential for manufacturing LWA.

## 3. Manufacture of Lightweight Aggregate Using Volcanic Mud and Paper Sludge as Additive

Volcanic mud and paper sludge were used to manufacture LWA under different sintering conditions. The feasibility of producing LWA was investigated using volcanic mud and mixed sludge containing volcanic mud and paper sludge. The mixed sludge was made with 10%, 20%, and 30% by weight replacements. The procedure followed for LWA production comprised jaw crushing, milling, and graining for the formation of raw pellets and firing for LWA sintering.

### 3.1. Preparation of Raw Pellets

**Figure 6:**
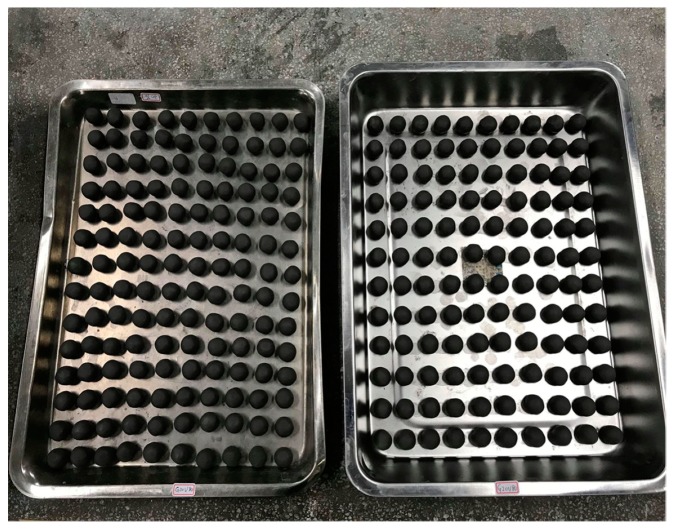
Raw pellets of mixed sludge.

Volcanic mud and mixed sludge (volcanic mud + paper sludge) were used individually to manufacture LWA. The collected raw materials were dewatered mechanically, and dehydrated in air until reaching the water content of 20–28%. The aggregate pellets were produced using plastic pelletization, that is, the materials after uniform distribution were added to water of different amounts which were measured based on the material’s ability to undergo plastic pelletization. The water-containing mixture of materials was made into spherical green pellets with a diameter of approximately 12 mm (Figure 6) by an extruder (Ko Tsao Co. Ltd., Taipei, Taiwan). Prior to the firing process, the green pellets were dried (at 105 °C for 24 h) to strengthen the pellets and prevent them from cracking during high-temperature sintering.

### 3.2. Sintering

To simulate the sintering conditions of existing mass production equipment, the heat treatment method used for the LWA sintering test involved a rapid, two-stage temperature rise. The main apparatus used in these experiments was a custom-made electrically heated oven consisting of two parts: a preheating oven and a sintering oven. The sintering was conducted by preheating the dried pellets in the preheating oven at a selected temperature for a certain period of time. The pellets were then transferred using a carrier to the sintering oven for the bloating and sintering reactions in atmospheric air at a high temperature for a soaking time. After the sintering process, the pellets were moved to a cooling room to cool down to room temperature. The temperature and soaking time for the sintering tests were as follows:

1. Preheating
  (1) Temperature: 500 °C, 600 °C, and 700 °C
  (2) Time: 5, 7, and 15 min
2. Sintering
  (1) Temperature: 950 °C, 1000 °C, 1100 °C, 1150 °C, 1200 °C, 1250 °C, and 1275 °C
  (2) Time: 2.5, 5, 7.5, 10, and 15 min

### 3.3. Lightweight Aggregate Property Test

Appropriate sintering conditions were investigated using the aforementioned sintering procedure and by observing the appearance and property of the resulting LWA to study the effects of using volcanic mud and paper sludge on LWA production. The required physical and mechanical properties, such as particle density, water absorption, and crushing strength, were tested in accordance with ASTM C29, ASTM C127, and GB2842, respectively. In addition, the microstructure of the sintered LWA was examined using a scanning electron microscope (JEOL, Tokyo, Japan).

## 4. Results and Discussion

Taking into consideration the reports of Huang and Wang [25] and Chen et al. [26] that investigated the manufacturing technologies of LWA from reservoir sediments and water treatment sludge, the authors of this research preliminarily adopted volcanic mud and mixed sludge (volcanic mud incorporating various percentages of paper sludge) to experimentally manufacture LWA with adequate operating conditions of preheating temperature, preheating time, sintering temperature, and sintering soaking time. The test results of the produced LWAs are summarized in Table 3 and Table 4.

**Figure 7:**
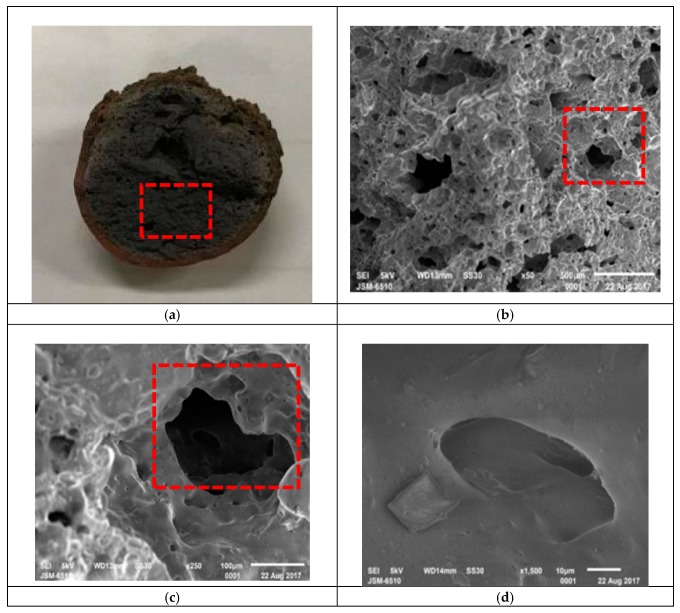
Cross-section and SEM micrographs of sintered LWA. (**a**) Cross-section; (**b**) SEM ×50; (**c**) SEM ×250; (**d**) SEM ×1500.

The experiment was performed with dried pellets in the oven using a sequential process. It started with a preheating temperature in the range of 500 to 700 °C for a preheating time of 5 to 15 min, followed by sintering at a temperature ranging from 950 to 1275 °C for a soaking time of 2.5 to 15 min. During the experiment, it was observed that the material of the raw pellet softened, started melting, and released gases. During this process, the green pellets were transformed into ceramic spherical granules containing a significant glassy phase with isolated and irregular pores. Figure 7 shows a typical round shape and cross-section of the sintered LWA with a hard ceramic shell and porous cores inside. The results of these investigations confirm the feasibility of manufacturing LWA from the volcanic mud and mixed sludge with the abovementioned ranges of preheating temperature, sintering temperature, and soaking time. However, it was also found in the experiments that the properties of the produced LWA were varied and depended upon the temperature and time used for the sintering tests. The following paragraphs further discuss how to interpret the effects of operating conditions on the properties of the produced LWAs.

### 4.1. Manufacturing Lightweight Aggregate with Volcanic Mud

As a result of chemical composition analysis, the composition of the volcanic mud can meet Riley’s limit of bloating material. Volcanic mud was used solely as raw material. Lightweight aggregates were produced with appropriately selected preheating temperatures, sintering temperatures, and soaking times. The produced LWAs were then tested for particle density, water absorption, and crushing strength. Using these results, the effects of operating conditions on the properties of the sintered LWA are discussed as follows:

(1) Effects of preheating temperature on the particle density and water absorption of produced LWA

**Figure 8:**
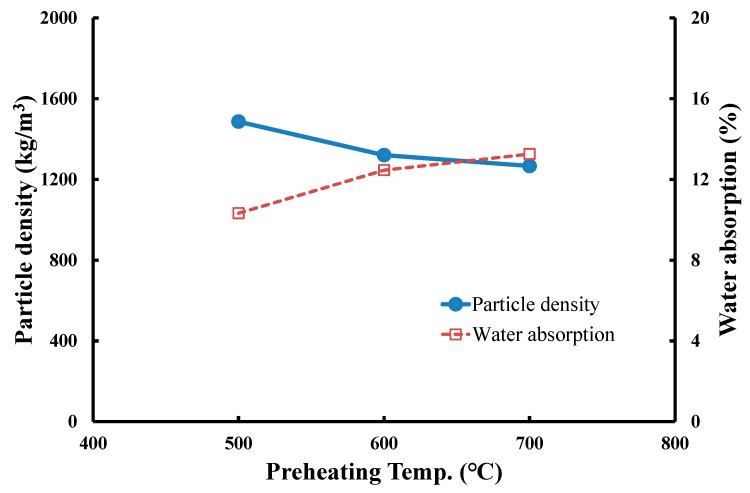
Effects of preheating temperature on the particle density and water absorption of LWA.

In the experiments, the operating conditions for LWA production were set as follows: (a) three preheating temperature levels of 500 °C, 600 °C, and 700 °C with a soaking time of 5 min and (b) a sintering temperature of 1200 °C with a soaking time of 5 min. The produced LWAs were tested for particle density and water absorption. Figure 8 shows the measured results. The particle density of LWA tends to decrease with the increase in preheating temperature, while the increase in preheating temperature leads to the increase in water absorption. Results show that when the preheating temperature increased from 500 to 700 °C, the particle density decreased from 1486 to 1267 kg/m^3^, and the water absorption increased from 10.3 to 13.3%. This is because enhancing the preheating temperature may induce more substances to develop gas, and that with a shorter sintering time (5 min), more volatilized gases may remain in the interior of pellet until the sintering stage. These gases form pores in the sintering LWA, expanding the size of the particles. Consequently, under the same conditions of sintering temperature (1200 °C), sintering time (5 min), and preheating time (5 min), the increase in preheating temperature from 500 to 700 °C may have an inverse relationship with the particle density but a direct proportion with the water absorption of LWA.

(2) Effects of preheating time on the particle density and water absorption of produced LWA

**Figure 9:**
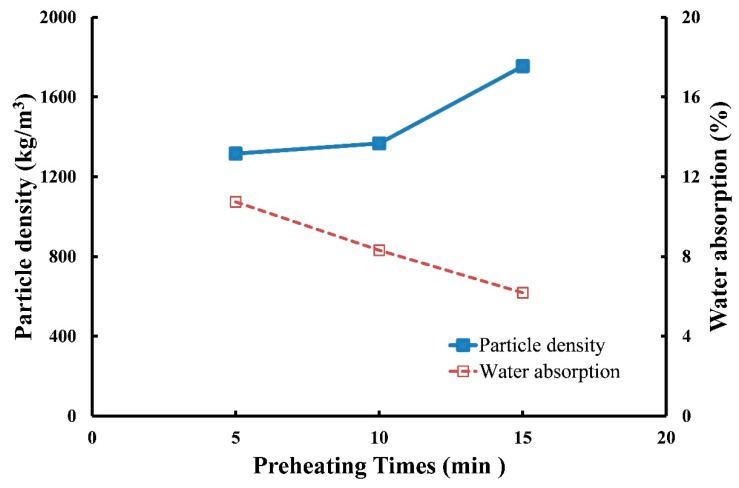
Effects of preheating time on the particle density and water absorption of LWA.

To manufacture LWA for testing, the operating conditions were set as follows: (a) a preheating temperature of 500 °C with three soaking times of 5, 10, and 15 min and (b) a sintering temperature of 1200 °C with a soaking time of 5 min. Figure 9 presents the test results for LWA particle density and water absorption. The particle density increased as preheating time increased, whereas the water absorption decreased as preheating time increased. The results revealed that when the preheating time increased from 5 to 15 min, the particle density increased from 1316 to 1755 kg/m^3^, and the water absorption decreased from 10.7 to 6.2%. This is because increasing the preheating time may simultaneously delay the volatilization duration of generated gas, affecting the expansion formation of pellets and resulting in a denser structure. Therefore, under the same sintering temperature (1200 °C), sintering time (5 min), and preheating temperature (500 °C) conditions, the increase in preheating time from 5 to 15 min may be directly proportional to the particle density but inversely related to the water absorption of LWA.

(3) Effects of sintering temperature on the particle density and water absorption of produced LWA

**Figure 10:**
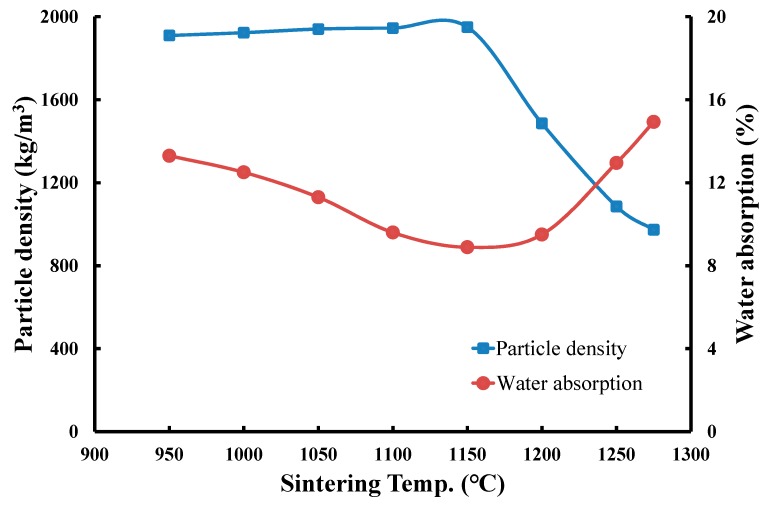
Effects of sintering temperature on the particle density and water absorption of LWA.

The operating conditions of LWA production were set as follows: (a) a preheating temperature of 500 °C with a soaking time of 5 min and (b) several sintering temperatures ranging from 950 to 1275 °C with a soaking time of 5 min. Figure 10 presents the test results of LWA particle density. At sintering temperatures ranging from 950 to 1150 °C, the particle density of the produced LWAs was in the range of 1910–1950 kg/m^3^ with a slight change of less than 2.5%. These results demonstrated that the glassy phase could not form in this temperature range (950–1150 °C) to trap the liberated gases and expand the raw pellets. When the temperature further increased from 1150 to 1275 °C, the particle density decreased gradually from 1950 to 970 kg/m^3^. This revealed that the pellets began to generate a glass-formed matrix at temperatures higher than 1150°C, eventually trapping the gases and resulting in the expansion of LWAs. Therefore, under the same sintering time (5 min), preheating temperature (500 °C), and preheating time (5 min) conditions, the particle density of LWAs may on average be approximately 1900–1950 kg/m^3^ at sintering temperatures from 950 to 1150 °C. However, by contrast, a further increase in temperature from 1150 to 1275 °C gradually reduces the particle density to approximately 970 kg/m^3^.

The measured results of the water absorption of LWAs, as shown in Figure 10, reveal that as the sintering temperature increased from 950 to 1150 °C, the water absorption decreased from 13.3 to 9.6%, indicating that the pellets adopted a denser structure as the temperature increased, resulting in a reduction of water absorption. When the sintering temperature further increased from 1150 to 1275 °C, the LWA water absorption was increased from 8.89 to 14.9%. This was because after the temperature increased to 1150 °C, the glassy phase formed to encapsulate the gases, leading to the expansion and production of pores. These connective pores provided paths for water to enter the LWA, thus increasing the water absorption. Therefore, under the same sintering time (5 min), preheating temperature (500 °C), and preheating time (5 min) conditions, the water absorption of LWA decreased from 13.3 to 8.89% with the increase in sintering temperature from 950 to 1150 °C, whereas after the increase in temperature from 1150 to 1275 °C, the water absorption increased from 8.89 to 14.9%, indicating that the produced LWA had the lowest water absorption at the sintering temperature of approximately 1150 °C.

(4) Effects of sintering time on the particle density and water absorption of produced LWA

**Figure 11:**
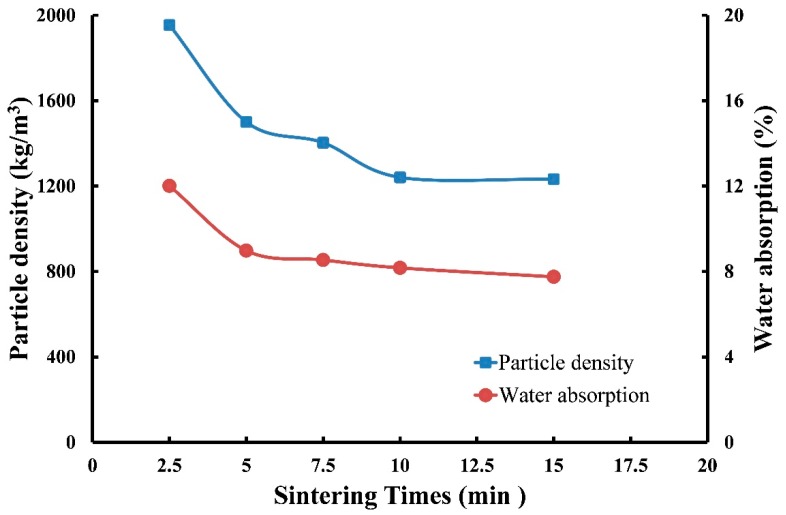
Effects of sintering time on the particle density and water absorption of LWA (sintering temperature at 1150 °C).

The operating conditions for LWA production were set as follows: (a) a preheating temperature of 500 °C with a soaking time of 5 min and (b) a sintering temperature of 1150 °C with five sintering times of 2.5, 5.0, 7.5, 10, and 15 min. Figure 11 presents the test results for the particle density of LWAs versus sintering time. The particle density produced for the sintering time of 2.5 min exhibited a high value of 1954 kg/m^3^. This density was reduced to 1501 kg/m^3^ for the sintering time of 5 min, and then it smoothly decreased to 1232 kg/m^3^ for 15 min. This result indicated that with a sintering time of 2.5 min, the pellets could not liberate sufficient gases for expansion during the sintering process, whereas 5 min was an adequate sintering time for producing qualified LWAs.

The measured results of the water absorption of LWAs illustrated in Figure 11 reveal a similar curve trend to that of the particle density. The water absorption of LWAs produced at the sintering time of 2.5 min had a relatively high value of 12.0%, which was greatly reduced to 8.89% at 5 min, and then also smoothly reduced to 7.75% at 15 min. This result was because a longer sintering time may produce more glass-formed material on the surface of the particle that is becoming a hard shell. On the basis of previously observed results, it was concluded that under the same sintering temperature (1150 °C), preheating temperature (500 °C), and preheating time (5 min) conditions, the particle density and water absorption of sintered LWAs exhibited a similar trend with sintering times from 2.5 to 15 min, as shown in Figure 11. At 2.5 min, particle density and water absorption had relatively high values of 1954 kg/m^3^ and 12.0%, respectively. At 5 min, both values reduced sharply to 1501 kg/m^3^ and 8.89%, respectively. After the sintering time was further increased from 5 to 15 min, the particle density decreased smoothly from 1501 to 1232 kg/m^3^, and the water absorption also decreased from 8.89 to 7.75%. Consequently, 5 min can be selected as an adequate sintering time for producing qualified LWA.

(5) Effects of operating conditions on the crushing strength of produced LWA

**Figure 12:**
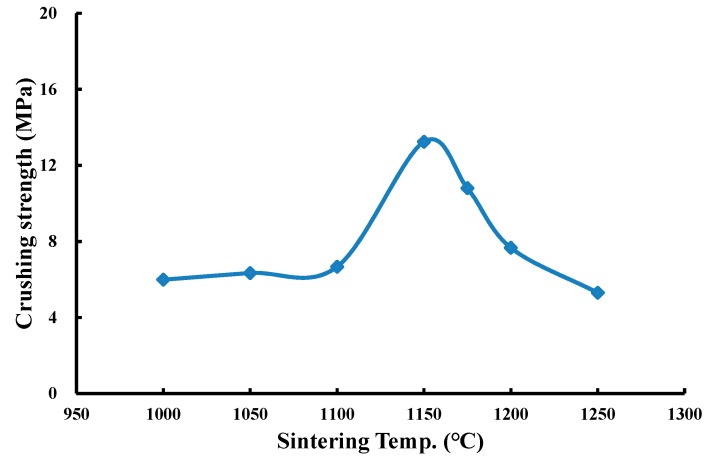
Effects of sintering temperature on the crushing strength of LWA.

Crushing strength is a representative property for the mechanical properties of LWA. During LWA production, operating conditions affect the interior pore structure of sintered LWA. The pore structure, including pore size, content, and distribution, may determine the crushing strength. To investigate the influences of operating conditions on the crushing strength of LWA, four sintering temperature levels (1000 °C, 1150 °C, 1175 °C, and 1200 °C) were selected with the same preheating temperature (500 °C), preheating time (5 min), and sintering time (5 min) to manufacture LWAs. The sintered LWAs were used for the crushing strength test. The results as shown in Figure 12 demonstrate that the crushing strength increased from 6.0 to 13.2 MPa as the sintering temperature climbed from 1000 to 1150 °C but then decreased from 13.2 to 5.3 MPa when the temperature further increased from 1150 to 1250 °C. The reason for the occurrence of the inverse strength turning point is that after the temperature exceeded 1150°C, more glass-formed material was generated on the surface of the pellets capable of encapsulating the gases and expanding the LWA, thus reducing the crushing strength.

### 4.2. Manufacturing Lightweight Aggregate with Mixed Sludge

Mixed sludge was used as the raw material for the LWA manufacturing experiment. The sludge was prepared by mixing the volcanic mud and paper sludge with three weight ratios (i.e., 90:10, 80:20, and 70:30). The raw pellets of mixed sludge were entered into the firing process with appropriately selected preheating temperatures, sintering temperatures, and soaking times. The produced LWAs were then tested for physical and mechanical properties. Measured results were adopted to investigate the correlation between sintering conditions and the properties of LWA.

**Figure 13:**
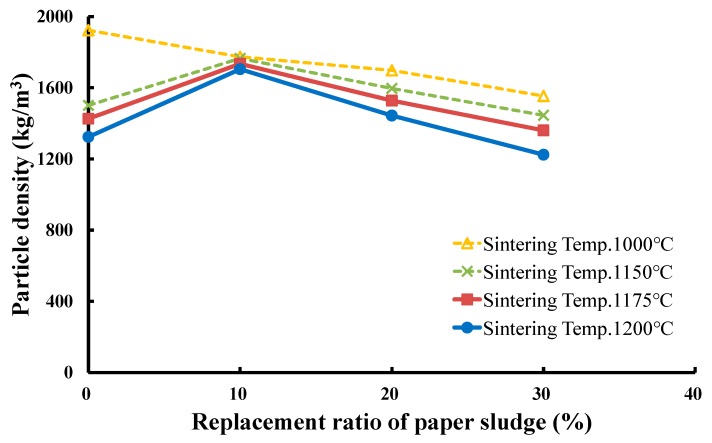
Effects of the replacement ratio of paper sludge on the particle density of LWA.

**Figure 14:**
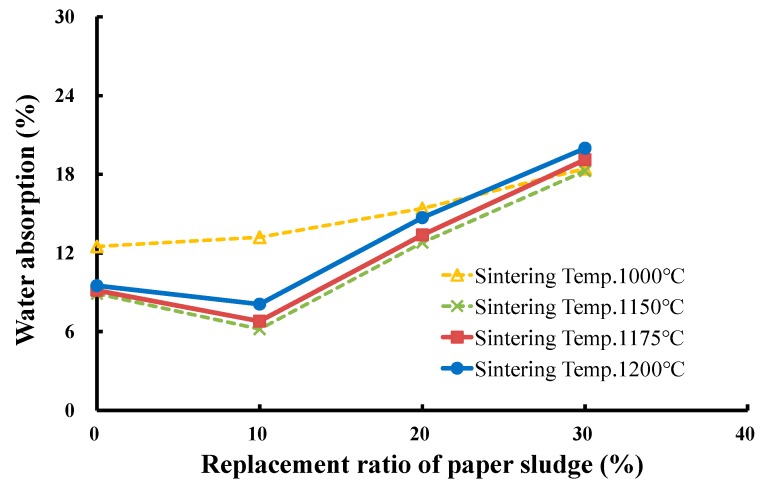
Effects of the replacement ratio of paper sludge on the water absorption of LWA.

**Figure 15:**
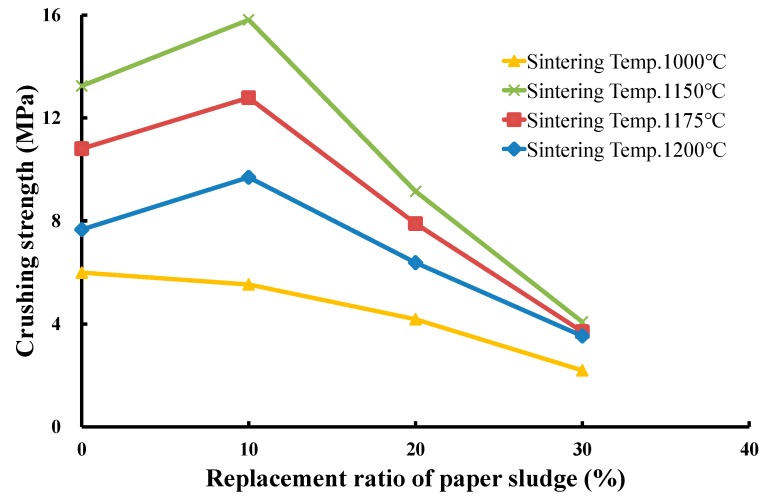
Effects of the replacement ratio of paper sludge on the crushing strength of LWA.

To produce LWAs in the experiment, the operating conditions were set as follows: (a) four sintering temperatures of 1000 °C, 1150 °C, 1175 °C, and 1200 °C with a soaking time of 5 min and (b) a preheating temperature of 500°C with a soaking time of 5 min. The mixed sludge with three replacement ratios (10%, 20%, and 30%) of paper sludge was successfully used to produce qualified LWAs. The produced LWAs were tested for particle density, water absorption, and crushing strength. Figure 13, Figure 14 and Figure 15 present the measured results of these properties for the three replacement ratios of paper sludge. The effects of the replacement ratio and sintering temperature on the properties of LWA are discussed in the following section.

(1) Effects of the replacement ratio of paper sludge on the particle density of produced LWA

Figure 13 shows that at the sintering temperature of 1000 °C, the particle density of the sintered LWAs decreased as the replacement ratio of paper sludge increased. A less glassy phase was formed on the surface of the particles at this temperature, which was unable to encapsulate the generated gases. However, increasing the replacement ratio of paper sludge led to an increase in the loss of ignition for the firing pellets. More gases were then liberated and volatilized away, resulting in a decrease in particle density. The particle density of LWA gradually decreased from 1923 kg/m^3^ with a 0% replacement ratio of paper sludge to 1774, 1698, and 1553 kg/m^3^, with 10%, 20%, and 30% replacement ratios of paper sludge, respectively.

When the sintering temperature increased to higher than 1150 °C, namely, 1175 °C and 1200 °C, the particle density of the produced LWA exhibited a similar varying tendency with the replacement ratio of paper sludge, as shown in Figure 13. First, the particle density increased from that of the 0% replacement ratio to a maximum value at the 10% replacement ratio, then inversely decreased gradually at the 30% replacement ratio. For instance, at the sintering temperature of 1175 °C, the particle density was 1425 kg/m^3^ for the 0% replacement ratio, 1734 kg/m^3^ for 10%, 1527 kg/m^3^ for 20%, and 1360 kg/m^3^ for the 30% replacement ratio. The reason for these unique results could be that when increasing the replacement ratio from 0 to 10%, the glass-formed matrix started to form on the particle surface during the sintering process. However, its stickiness was not yet sufficient to encapsulate the gases to expand the pellets, resulting in a denser structure and the increase in particle density. On the contrary, when the replacement ratio increased from 10 to 30%, based on the increase rate of the fluxing in the raw pellet, more glassy phases formed on the particle surface, which trapped the gases expanding the pellet and thus decreased the particle density. As a whole, the paper sludge reacting as fluxing in the mixed sludge may have affected the sintering performance, particularly at sintering temperatures exceeding 1150 °C. With the aid of the fluxing, the pellet may have formed a glassy phase on the surface at a lower temperature, consequently expanding the produced LWA and in turn decreasing the particle density. LWA exhibited a maximum particle density of approximately 1700 kg/m^3^ at the paper sludge replacement ratio of 10%.

(2) Effects of the replacement ratio of paper sludge on the water absorption of produced LWA

Figure 14 illustrates that the relationships between the water absorption of the produced LWAs and the paper sludge replacement ratio of mixed sludge for various sintering temperatures have a similar but inverse tendency to the particle density of LWA versus the paper sludge replacement ratio. These results indicate that at the sintering temperature of 1000 °C, the water absorption of LWAs increased as the replacement ratio of paper sludge increased; the value of water absorption increased from 12.5% with the 0% replacement ratio up to 13.2%, 15.4%, and 18.4% with 10%, 20%, and 30% replacement ratios of paper sludge, respectively. In addition, at sintering temperatures higher than 1150 °C, namely, 1175 °C and 1200 °C, the water absorption of LWAs first decreased from that of the 0% replacement ratio to that of the minimum value of the 10% replacement ratio, then inversely increased gradually to that of the 30% replacement ratio. For example, at the sintering temperature of 1175 °C, the water absorption was 9.2% for the 0% replacement ratio, 6.8% for 10%, 13.4% for 20%, and 19.1% for the 30% replacement ratio. The reason for these results could be similar to that regarding the particle density, except that the water absorption and particle density displayed inverse effects on the property of LWA. Thus, the water absorption of the produced LWAs had a minimum value of approximately 15% at the 10% replacement ratio of paper sludge in mixed sludge for sintering temperatures higher than 1150 °C, whereas the water absorption may increase to approximately 19% at the replacement ratio of 30%.

(3) Effect of the replacement ratio of paper sludge on the particle cylindrical crushing strength of produced LWA

Figure 15 illustrates the relationship between the measured particle cylindrical crushing strength of the produced LWAs and the replacement ratio of paper sludge in the mixed sludge. At the sintering temperature of 1000 °C, the crushing strengths of the LWAs gradually decreased as the replacement ratio of paper sludge increased and were all lower than those of the LWAs produced at the higher sintering temperatures of 1150 °C, 1175 °C, and 1200 °C. This is because less glass-formed material was formed on the surface of the particles at 1000 °C, and the increase in the replacement ratio of paper sludge liberated more gases and volatilized them away, resulting in more highly proliferous particles without hard ceramic shells, which were thus weak. At this temperature, the particle cylindrical crushing strength gradually decreased from 6.0 MPa for the 0% replacement ratio of paper sludge to 5.5, 4.2, and 2.2 MPa for the 10%, 20%, and 30% replacement ratios of paper sludge, respectively.

When the sintering temperature increased to higher than 1150 °C, namely, 1175 °C and 1200 °C, the relationship between the crushing strength of the produced LWAs and the paper sludge replacement ratios of mixed sludge, as shown in Figure 15, exhibited a similar tendency to that of the particle density of LWA versus paper sludge replacement ratio. The crushing strength of the LWAs first increased from that of the 0% replacement ratio to that of a maximum value at the 10% replacement ratio, then inversely decreased to that of the 30% replacement ratio. For example, at a sintering temperature of 1175 °C, the crushing strength was 10.8 MPa for the 0% replacement ratio, 12.8 MPa for 10%, 7.9 MPa for 20%, and 3.7 MPa for the 30% replacement ratio. The reason for these results also relates to the particle density because a higher particle density could lead to a higher LWA crushing strength. Consequently, it can be concluded that at the sintering temperature of 1000 °C, the crushing strength of the produced LWA decreased with the replacement ratio of paper sludge, varying in the range between 5.5 and 2.2 MPa. However, at sintering temperatures exceeding 1150 °C, for example, at 1175 °C, the crushing strength of the LWAs first increased from 10.8 MPa for the 0% replacement ratio to a maximum value of 12.8 MPa for the 10% replacement ratio, and then inversely decreased gradually to 3.7 MPa for the 30% replacement ratio.

## 5. Conclusions

The volcanic mud collected from Pingtung County in Taiwan can be used in LWA production, even without any gas-releasing additives. The paper sludge collected from the paper factory in Hualien County, Taiwan can be used feasibly as a fluxing agent additive incorporated into volcanic mud to produce LWAs. Both of the materials can be used as primary resource material for lightweight aggregate that can not only achieve technical benefits, but also result in good social and ecological benefits.

The LWAs manufactured using volcanic mud with the operating conditions of a preheating temperature from 500 to 700 °C with a preheating time from 5 to 15 min and a sintering temperature from 950 to 1275 °C with sintering times from 2.5 to 15 min can exhibit particle density ranging from 1950 to 933 kg/m^3^, water absorption ranging from 0.18 to 13.2%, and crushing strength ranging from 5.3 to 13.2 MPa. A preheating temperature of 500 °C with a preheating time of 5 min is proposed as suitable conditions for LWA production. Combined with these preheating conditions, a sintering temperature of 1150 °C with a sintering time of 5 min could produce LWA possessing the lowest water absorption and the highest crushing strength.

LWAs manufactured using mixed sludge (volcanic mud and paper sludge) with the operating conditions of a preheating temperature of 500 °C with a preheating time of 5 min and a sintering temperature from 1000 to 1200 °C with a sintering time of 5 min, for the paper sludge content of 10–30% by weight of mixed sludge, can exhibit particle density from 1923 to 1224 kg/m^3^, water absorption from 6.2 to 20%, and crushing strength from 2.2 to 15.81 MPa. The particle density and crushing strength of the produced LWAs decrease with the increase in the replacement ratio of the paper sludge. On the contrary, the water absorption of LWA increases with the increase in the replacement ratio of paper sludge.

## Figures and Tables

**Table 1:** Physical test results of volcanic mud and paper sludge.

Material	Specific Gravity	PI (%)	D_50_	Ingredients (wt %)		
				Sand	Silt	Clay
Volcanic mud	2.73	8–10	0.0715	1	47	53
Paper sludge	1.03	10–15	0.0998	0	43	57

Note: PI, plasticity index; D_50_, median diameter.

**Table 2:** Chemical composition of volcanic mud and paper sludge.

Material	Chemical Composition (%)								
	SiO_2_	Al_2_O_3_	Fe_2_O_3_	K_2_O	MgO	Na_2_O	CaO	LOI	Total
Volcanic mud	64.2	15.3	6.30	2.60	2.50	1.71	1.52	5.67	100.00
Paper sludge	3.95	1.84	3.20	0.82	6.97	3.29	41.27	38.54	100.00
Riley’s limit of bloating material	53–79	12–26	Fluxing = 8–24					-	-

**Table 3:** Properties of lightweight aggregates (LWAs) with volcanic mud.

Preheating		Sintering		Particle Density (kg/m^3^)	Water Absorption (%)
Temperature (°C)	Time (min)	Temperature (°C)	Time (min)		
500	5	1200	5	1486	10.32
600				1320	12.46
700				1267	13.26
500	5	1200	5	1316	10.74
	10			1368	8.31
	15			1755	6.18
500	5	950	5	1909	13.30
		1000		1923	12.50
		1050		1941	11.30
		1100		1950	9.60
		1150		1501	8.89
		1200		1323	9.50
		1250		1086	12.95
		1275		973	14.93
500	5	1150	2.5	1955	12.01
			5.0	1501	8.89
			7.5	1403	8.54
			10	1241	8.17
			15	1232	7.75

**Table 4:** Properties of LWAs with mixed sludge.

Preheating		Sintering		Replacement Ratio of Paper Sludge (%)	Particle Density (kg/m^3^)	Water Absorption (%)	Crushing Strength (MPa)
Temperature (°C)	Time (min)	Temperature (°C)	Time (min)				
500	5	1000	5	0%	1923	12.5	5.99
				10%	1774	13.2	5.53
				20%	1698	15.4	4.18
				30%	1553	18.4	2.20
500	5	1150	5	0%	1501	8.9	13.24
				10%	1765	6.2	15.81
				20%	1596	12.8	9.15
				30%	1445	18.3	4.08
500	5	1175	5	0%	1426	9.2	10.80
				10%	1734	6.8	12.79
				20%	1527	13.4	7.90
				30%	1360	19.1	3.70
500	5	1200	5	0%	1324	9.5	7.67
				10%	1704	8.1	9.70
				20%	1444	14.7	6.38
				30%	1224	20.0	3.53
